# Analysis of deep tissue hypersensitivity to pressure pain in professional pianists with insidious mechanical neck pain

**DOI:** 10.1186/1471-2474-12-268

**Published:** 2011-11-24

**Authors:** Marcela Linari-Melfi, Irene Cantarero-Villanueva, Carolina Fernández-Lao, César Fernández-de-las-Peñas, Rafael Guisado-Barrilao, Manuel Arroyo-Morales

**Affiliations:** 1 Forexpla Prevention Department. Real Conservatorio Superior de Música de Madrid. Escuela Superior de Canto de Madrid, Madrid, Spain; 2 Department of Physical Therapy, Universidad Granada, Spain; 3 Department of Physical Therapy, Occupational Therapy, Rehabilitation and Physical Medicine, Universidad Rey Juan Carlos, Alcorcón, Madrid, Spain; 4 Laboratory of Esthesiology, Universidad Rey Juan Carlos, Alcorcón, Madrid, Spain; 5 Department of Nursing, Universidad de Granada, Spain

## Abstract

**Background:**

The aim of this study was to investigate whether pressure pain hyperalgesia is a feature of professional pianists suffering from neck pain as their main playing-related musculoskeletal disorder.

**Methods:**

Twenty-three active expert pianists, 6 males and 17 females (age: 36 ± 12 years) with insidious neck pain and 23 pianists, 9 males and 14 females (age: 38 ± 10 years) without neck pain the previous year were recruited. A numerical pain rate scale, Neck Disability Index, hand size and pressure pain thresholds (PPT) were assessed bilaterally over the C5-C6 zygapophyseal joint, deltoid muscle, the second metacarpal and the tibialis anterior muscle in a blinded design.

**Results:**

The results showed that PPT levels were significantly decreased bilaterally over the second metacarpal and tibialis anterior muscles (P < 0.05), but not over C5-C6 zygapophyseal joint and deltoid muscle (P > 0.10), in pianists with neck pain as compared to healthy pianists. Pianists with neck pain had a smaller (P < 0.05) hand size (mean: 181.8 ± 11.8) as compared to pianists without neck pain (mean: 188. 6 ± 13.1). PPT over the tibialis anterior muscles was negatively correlated with the intensity of neck pain.

**Conclusions:**

Our findings revealed pressure pain hypersensitivity over distant non-symptomatic distant points but not over the symptomatic areas in pianists suffering from neck pain. In addition, pianists with neck pain also had smaller hand size than those without neck pain. Future studies are needed to further determine the relevance of these findings in the clinical course of neck pain as playing-related musculoskeletal disorder in professional pianists.

## Background

Work-related musculoskeletal disorders (WMSD) cause pain, disability, and loss if employment for workers enrolled in several occupations [1] As playing of an instrument is an example of work, [2] playing-related musculoskeletal disorder (PRMD) is the proper term related to music-specific work-related musculoskeletal disorder. In fact, PRMD is defined as "...pain, weakness, lack of control, numbness, tingling, or other symptom that interfere with musicians' ability to play the instrument at the level he/she is accustomed to..." [3]

PRMDs area a recognized problem amongst instrumental musicians and include overuse problems, e.g., tendinitis and peripheral nerve entrapment syndromes. It seems that the prevalence of PRMDs in musicians is consistent with the prevalence of work-related musculoskeletal disorders for other workers [4]. In a systematic review, Bragge et al. reported a wide range in prevalence rates for PRMD from 26% to 93% in pianist [5]. Although pianists are prominent in data regarding prevalence of PRMDs, there is poor understanding of piano-specific risk factors associated with particular PRMD. A recent study revealed that the prevalence of neck pain (29.3%) was the most common PRMD, and similarly to upper limb pain (ranging from 20% to 30.4%) experienced by piano students [6]. As economic burden of neck pain involves high annual compensation costs, [7] studies investigating etiological mechanisms in pianists with neck pain are needed.

It has been an increasing interest for impairments in nociceptive pain processing in the last decade in patients with neck pain. In fact, impairments in nociceptive gain by have been found in individuals with whiplash-associated neck pain [8] and idiopathic neck pain [9]. These studies evaluated pressure pain thresholds [10] and reported that patients with insidious neck pain exhibited pressure pain hypersensitivity (i.e., lower pressure pain thresholds, PPT) exclusively over the symptomatic cervical area, whereas patients with whiplash-associated neck pain also exhibited lower PPT over non-symptomatic areas such as the tibialis anterior muscle [11]. Scott et al concluded that insidious mechanical neck pain reflects segmental local sensitization whereas whiplash-associated neck pain reflects an augmented central pain processing mechanism, i.e., central sensitization [11].

To the best of the authors' knowledge, no study has previously investigated the presence of generalized deep tissue pressure hyperalgesia in professional pianists with neck pain as their main PRMD. The aim of the present study was to investigate whether pressure pain hyperalgesia is a feature of professional pianists suffering from neck pain as their main PRMD.

## Methods

### Participants

Active expert pianists from Madrid Music Academy were recruited. The current study focuses on insidious neck pain as main playing-related musculoskeletal disorder. We included pianists with current insidious neck pain and pianists without neck pain the previous years as control group. Insidious neck pain was defined as generalized neck or shoulder pain of mechanical characteristics provoked by neck postures, neck movement, or palpation of the cervical muscles. Participants were excluded if exhibited any of the following criteria: 1, previous surgery and/or steroid injections in the upper quadrant; 2, whiplash cervical or neck surgery; 3, history of wrist or arm trauma; 4, symptoms in any different place than the neck-shoulder area, for instance, in the hand; or, 5, fibromyalgia syndrome [12].

Self-reported handedness, degree of the course and relative year attended, age at start, the possibility to adjust the chair height, number of hours/day and hours/week spent in individual practice, frequency and duration of the breaks, and frequency and duration of preliminary technical exercises were recorded. Participants were asked to indicate whether they believe that "a certain amount of pain is acceptable when attempting to overcome technical difficulties" ("No pain, no gain" criterion). The study was approved by the Ethics committee at Granada University and informed consent was obtained from all participants.

### Self-reported measures

An 11-point numerical pain rate scale (NPRS, 0: no pain; 10: maximum pain) was used to assess the current level of neck pain and shoulder pain. The NPRS has been demonstrated to be a reliable and valid instrument to assess pain intensity [13]. Patients also completed the Neck Disability Index (NDI) to assess self-perceived disability. The NDI consist of 10 questions measured on a 6-point scale (0: no disability; 5: full disability) [14]. The numeric score for each item is summed for a score varying from 0 to 50, where higher scores reflect greater disability. The NDI is a reliable and valid outcome of disability in neck pain [15,16]. Macdemid et al found that studies investigating reliability of the NDI showed intra-class correlation coefficients ranging from 0.50 to 0.98, suggesting that the NDI has sufficient support and usefulness to be the most commonly used self-report measure for neck pain [17]. Finally, piano players traced the outline of their dominant hand in a rest position (minimal abduction angle) on a graph paper. Hand length, breadth and index were evaluated by drawing lines and classified according to Wagner percentiles. [18].

### Pressure Pain Threshold Assessment

An electronic algometer (Somedic AB, Sweden) was used to determine pressure pain thresholds (PPT: minimal amount of pressure where a sensation of pressure first changes to pain) [19]. The pressure was applied approximately at a rate of 30 kPa/sec, with the algometer placed perpendicular to the application point. Participants were instructed to press switch when the sensation changed from pressure to pain. The mean of 3 trials (intra-examiner reliability) was calculated and used for the main analysis. A 30-s resting period was allowed between each measure. The reliability of pressure algometry has been found to be high (ICC: 0.91, 95% CI 0.82-0.97) [20].

All participants had abstained from any kind of general exercise the previous day and were not allowed to take analgesics or muscle relaxant through the 72 h prior to the examination. Participants attended a preliminary session for familiarization with PPT assessment. PPT levels were measured bilaterally over the articular pillar of C5-C6 zygapophyseal joint, the deltoid muscle, the second metacarpal and the tibialis anterior muscle by an assessor blinded to the participant condition. The order of assessment was randomized between participants.

### Sample Size Determination

The sample size determination was done with an appropriate software (Tamaño de la Muestra, 1.1^©^, Spain). The determinations were based on detecting significant differences of 20% on PPT levels over each point between both groups [21] with an alpha level of 0.05, and a desired power of 80%. This generated a sample size of at least 16 participants per group.

### Statistical Analysis

Data were analysed with the SPSS statistical package (19.0 Version). Results are expressed as mean ± standard deviation and 95% confidence interval (95% CI). The Kolmogorov-Smirnov test was used to analyse the normal distribution of the variables (P > 0.05). Since quantitative data showed a normal distribution, parametric tests were used. Demographic characteristics of both study groups were compared using unpaired Student t-test for quantitative data and χ^2 ^tests of independence for categorical data. A two-way ANOVA test was used to evaluate the differences in PPT levels assessed over each point (C5-C6 joint, deltoid muscle, second metacarpal, tibialis anterior) with side (dominant/non-dominant) as within-subjects factor and group (neck pain or healthy) as between-subjects factor. Finally, the Pearson correlation test (r) was used to analyse the association between PPT, pain intensity (NPRS), and self-reported disability (NDI) in those pianist with insidious neck pain. The statistical analysis was conducted at a 95% confidence level. A P-value less than 0.05 was considered statistically significant.

## Results

### Demographic data of the participants

Twenty-three active expert pianists, 6 males and 17 females, with insidious neck pain and 23 expert pianists, 9 males and 14 females without pain the previous year were recruited. Overall, participants had 27.4 years of piano lessons, with 25.7 hours/week of piano lessons and 98.15 uninterrupted minutes of piano playing/day (mean ± SD: 98.2 ± 67.6 minutes uninterrupted of piano practice). All participants were university education level and had received prizes at domestic or international classic piano competitions. Seventy-six percent (76%) were right-handed, and the remaining 24% were left-handed. No differences in demographic (age, height, weight, BMI) and technical features (years of piano lessons, hours per week of piano lessons and minutes of piano playing per day) between groups were found (Table 1).

**Table 1:** Demographic data of professional pianists with insidious neck pain and pianists without neck pain

	Pianists with neck pain	Pianists without neck pain	Significance
**Gender (male/female)**	6/17	9/14	χ^2 ^= 2.193; P = 0.459

**Age (years)**	36 ± 12	38 ± 10	t = 0.956; P = 0.463

**Height (kg.)**	65 ± 13	68 ± 12	t = 0.838; P = 0.407

**Weight (cm.)**	169 ± 9	172 ± 8	t = 0.978; P = 0.433

**BMI (kg/cm^2^)**	22.6 ± 3.3	23.1 ± 4.3	t = 0.497; P = 0.622

**Years of piano playing**	26 ± 11	29 ± 12	t = 0.955; P = 0.345

**Hours/week of piano lessons**	26 ± 10	28 ± 12	t = 0.969; P = 0.324

**Minutes of piano playing/day**	103 ± 84	93 ± 47	t = 0.486; P = 0.629

Values are expressed as mean ± standard deviation

Within the neck pain group, the mean duration of neck pain history was 4.4 ± 2.1 months, the mean intensity (NPRS) of neck pain was 3.5 ± 2.9, the mean intensity of shoulder pain was 4.1 ± 2.7, and the mean NDI was 8.2 ± 5.4. A significant positive correlation between current level of neck pain and disability (r = 0.667; P < 0.001) was found: the higher the intensity of neck pain, the higher the self-reported disability. In addition, a significant positive correlation between neck pain intensity and minutes of piano playing/day (r = 0.481; P = 0.020) was also found: the higher the minutes of piano playing per day, the higher the intensity of neck pain.

Finally, pianists reporting insidious neck pain had a smaller (t = 2.851; P = 0.047) hand size (mean: 181.8 ± 11.8) as compared to pianists without neck pain (mean: 188. 6 ± 13.1).

### Pressure pain sensitivity

The intra-examiner repeatability of PPT readings over the C5-C6 zygapophyseal joint, deltoid muscle, second metacarpal and tibialis anterior muscle was 0.91, 0.89, 0.93 and 0.92, respectively whereas the SEM was 4.5, 6.7, 6.5 and 7.8 kPa, respectively.

**Figure 1:**
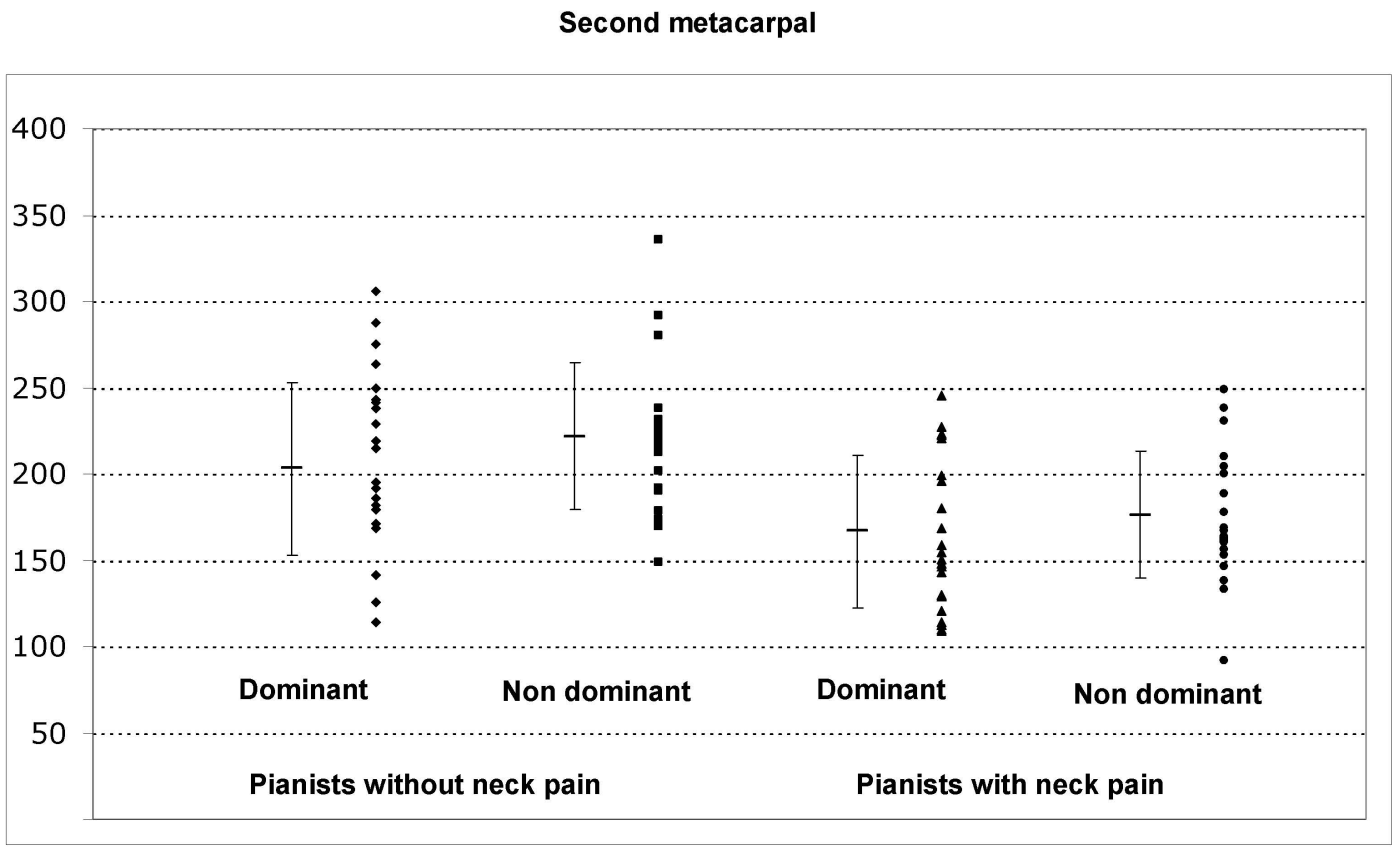
**Pressure pain thresholds (kPa) over the second metacarpal in pianists with neck pain and those without neck pain**. The horizontal bar represents the mean value and the error bars the standard deviation.

**Figure 2:**
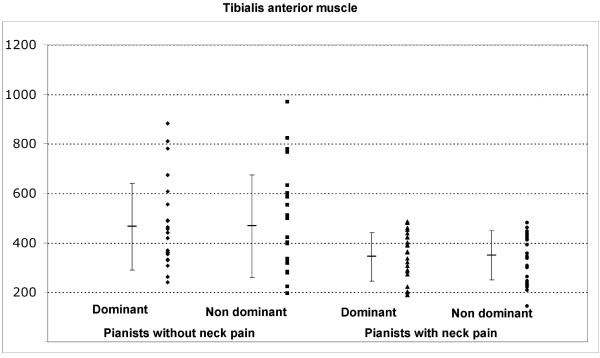
**Pressure pain thresholds (kPa) over the tibialis anterior muscle in pianists with neck pain and those without neck pain**. The horizontal bar represents the mean value and the error bars the standard deviation # indicates significant difference in PPT between patients and controls.

The ANOVA revealed significant differences between groups, but not side, for PPT over the second metacarpal (group: F = 10.898; P < 0.001; side: F = 0.1328; P = 0.252, Figure 1), and tibialis anterior muscle (group: F = 4.4.93; P = 0.041; side: F = 0.024; P = 0.878, Figure 2). Pianists reporting neck pain exhibited bilateral lower PPT over the second metacarpal (P < 0.001) and tibialis anterior muscles (P < 0.05) than those without neck pain. No significant differences between groups and sides for PPT over the C5-C6 zygapophyseal joint (group: F = 2.914; P = 0.091; side: F = 0.239; P = 0.626), and deltoid muscle (group: F = 0.600; P = 0.441; side: F = 0.134; P = 0.715) were found. Table 2 shows PPT assessed over the C5-C6 zygapophyseal joint, the deltoid muscle, the second metacarpal and the tibialis anterior muscle for both sides on each group.

**Table 2:** Differences in pressure pain thresholds (kPa) over c5-c6 zygapophyseal joint, deltoid muscle, second metacarpal and tibialis anterior muscles between professional pianists with insidious neck pain and pianists without neck pain

	C5-C6 joint	Deltoid muscle	Second metacarpal*	Tibialis anterior*
**Professional pianists with insidious neck pain**				
**Dominant**	208.8 ± 62.2 (180.0-237.7)	256.9 ± 166.1 (200.6-313.2)	166.7 ± 58.9 (142.0-191.3)	343.4 ± 97.8 (301.1-385.6)
**Non-dominant**	195.1 ± 55.8 (166.3-223.9)	256.2 ± 156.2 (199.9-312.5)	176.2 ± 45.1 (151.6-200.9)	349.4 ± 130.4 (293.5-406.2)
**Professional pianists without insidious neck pain**				
**Dominant**	227.3 ± 78.1 (197.8-256.7)	288.9 ± 114.1 (231.4-346.5)	202.9 ± 61.4 (178.2-227.6)	465.7 ± 180.2 (381.9-549.6)
**Non-dominant**	226.7 ± 79.3 (197.3-256.1)	268.5 ± 89.5 (211.1-326.2)	221.9 ± 69.1 (197.3-246.6)	467.7 ± 215.9 (384.0-551.7)

Values (kPa) are expressed as mean ± standard deviation (95% confidence interval)

* Significant differences between both groups controls (2-two way ANOVA test)

### Relationship between pressure pain sensitivity and neck pain

Finally, significant negative correlations between intensity of neck pain and PPT over both tibialis anterior muscles (dominant: r = -0.473; P = 0.020; non-dominant: r = -0.479; P = 0.021) were found: the higher the intensity of neck pain, the lower the bilateral PPT over the tibialis anterior muscles.

## Discussion

This is the first study investigating the presence of pressure pain sensitivity in pianists with neck pain as their main PRMD. The main finding of the present study was a bilateral decrease in PPT over non-symptomatic distant points, the second metacarpal and tibialis anterior muscles, but not over the symptomatic areas, the cervical spine and deltoid muscle, as compared to pianists without neck pain. Additionally, the decrease in PPT levels over the tibialis anterior muscle was associated with neck pain intensity. Finally, we also found that pianists presenting with neck pain had a small hand size than those without neck pain.

Prushansky et al. [21] established that differences between around 20%-25% are required to indicate a true clinical difference in PPT, at least in the cervical spine. In the current study, differences in mechanical sensitivity over the second metacarpal and the tibialis anterior muscle were superior to this value. In fact, current results were highly surprising as we showed that pianists with neck pain exhibit lower PPT levels over non-symptomatic points and normal over symptomatic areas. The presence of pressure pain hypersensitivity in distant pain-free areas indicates sensitization of the central nervous system in pianists suffering from neck pain; however, the absence of pressure hyper-sensitivity over the symptomatic areas makes this assumption inconclusive. This was an unexpected finding as previous studies have found the presence of lower PPT over the cervical spine in patients with insidious neck pain [11] or whiplash-associated neck pain [8]. Nevertheless, as central sensitization is a dynamic condition influenced by multiple factors including the activity of peripheral nociceptive inputs, [22] it may be that different factors are involved in our results. The existence of sensitization mechanisms in local pain syndromes suggests that sustained peripheral noxious input to the central nervous system play a role in the maintenance of central sensitization. In fact, in the current study, PPT over the tibialis anterior muscles was negatively associated with intensity of neck pain supporting this hypothesis. Again, the absence of mechanical hypersensitivity over the cervical spine was unexpected and deserves further research. It is possible that the fact that most pianists accept the "*no pain, no gain criterion" *can exert a cognitive influence on pressure pain sensitivity over the cervical spine. In addition, the presence of neck pain in pianists has been associated with high levels of static contraction, long periods of static load or forced postures occurring during playing. It is possible that professional pianists with neck pain adopt different strategies in the cervical spine to decrease tension within the neck muscles. Therefore, it is possible that the assessment of pressure pain sensitivity over C5-C6 zygapophyseal joint would be not the best option for this particular population. Future studies are clearly needed to further confirm these findings.

We also found that professional pianists with neck pain had a small hand size as compared to those without neck pain. Small hand size is the only risk factor that seems to be clearly associated with PRMDs in professional pianists [5]. It is possible that those pianists with small hands need more effort or induce greater physical demands on their upper extremities during playing promoting overload of the cervical spine structures.

We should recognise some limitations of the study. First, we included a relative small sample size. Larger studies with greater sample sizes are needed to permit a more generalized interpretation of our results. Further, it would be interesting to include other somato-sensory tests, such as vibration or thermal sensitivity, to investigate nociceptive pain processing in professional pianists with PRMD. Secondly, pressure pain sensitivity can be influenced by some psychological factors, e.g., depression or anxiety, or cognitive behaviours. Future studies should include these potential factors.

## Conclusion

This is the first study revealing the presence of pressure pain hypersensitivity in professional pianists with neck pain. Pianists with neck pain showed a bilateral decrease in PPT levels over non-symptomatic distant points, but not over the symptomatic areas, as compared to pianists without neck pain. Pianists with neck pain also had a smaller hand size than those without neck pain. Future studies are now needed to determine the clinical significance of these findings.

## Competing interests

The authors declare that they have no competing interests.

## Authors' contributions

MLM, ICV and CFL, carried out the musculoskeletal outcomes measurements, participated in the study design and drafted the manuscript. CFDLP, RGB participated in the design of the study, performed the statistical analysis and drafted the manuscript. MAM conceived of the study, and participated in its design and coordination and helped to draft the manuscript. All authors read and approved the final manuscript.

## Pre-publication history

The pre-publication history for this paper can be accessed here:

http://www.biomedcentral.com/1471-2474/12/268/prepub
